# Experiences and perceptions of lay bystanders responding to out-of-hospital cardiac arrest: A qualitative systematic review

**DOI:** 10.1097/MD.0000000000050530

**Published:** 2026-09-04

**Authors:** Shuyu Zhan, Xiaojiao Jiang, Mei Yang, Maodan Liu, Hanting Liu, Shisi Li

**Affiliations:** a Department of Cardiovascular Surgery, The Second Affiliated Hospital of Army Medical University: Xinqiao Hospital, Chongqing, China.

**Keywords:** cardiopulmonary resuscitation, lay bystander, out-of-hospital cardiac arrest, qualitative systematic review

## Abstract

**Background:**

To synthesize the experiences and perceptions of lay bystanders before, during, and after responding to out-of-hospital cardiac arrests.

**Methods:**

Seven English and Chinese databases were searched from their inception to July 31, 2026, supplemented with citation tracking. Two reviewers independently selected studies, appraised methodological quality using the Joanna Briggs Institute (JBI) critical appraisal checklist for qualitative research, and extracted the findings and supporting illustrations. Unequivocal and credible findings were synthesized using JBI meta-aggregation, and confidence was assessed using confidence in the evidence from reviews of qualitative research.

**Results:**

Eight studies involving 209 participants were included. Thirty-three findings were grouped into 12 categories and 4 were synthesized findings concerning the motivators and inhibitors of response, recognition and appraisal of out-of-hospital cardiac arrest, conditions facilitating or impeding cardiopulmonary resuscitation and automated external defibrillator use, and post-event psychological responses and adjustment. Confidence in the evidence from reviews of qualitative research was moderate for the 1st 3 synthesized findings, and low for the 4th.

**Conclusion:**

Bystander response is a continuum that extends from event recognition and action to post-event adjustment. Out-of-hospital cardiac arrest systems should provide realistic preparations, clear on-scene support, proportionate information, and psychological support after an event.

## 1. Introduction

Out-of-hospital cardiac arrest (OHCA), characterized by the abrupt cessation of cardiac mechanical activity and effective circulation outside the hospital, is a major public health emergency.^[1]^ OHCA is highly time-sensitive, and survival depends on early recognition, prompt activation of emergency medical services, high-quality cardiopulmonary resuscitation (CPR), and early use of an automated external defibrillator (AED).^[2]^ Because professional emergency responders are seldom able to reach the scene immediately, lay bystanders are often the 1st to activate a chain of survival. However, their presence does not necessarily translate into timely and effective action. A study conducted in Pakistan reported that a bystander was present in nearly 90% of OHCA events; however, only 2.3% of patients received bystander CPR,^[3]^ indicating a substantial gap between witnessing an event and intervening.

Previous OHCA research has primarily focused on the rates of bystander CPR and AED use, emergency response times, and patient survival, with comparatively limited attention paid to rescuer experiences. The American Heart Association has emphasized that bystanders are integral participants in OHCA systems of care, and that their training, involvement at the scene, and related experiences warrant systematic attention.^[4]^ Existing evidence suggests that the initiation of CPR depends not only on knowledge and skills but also on interacting psychological, behavioral, and situational factors.^[5,6]^ Much of this evidence is derived from quantitative or mixed-methods research and therefore cannot fully explain how bystanders interpret real events or make sense of their resuscitation experiences.

Qualitative research^[7]^ can provide in-depth accounts of participants’ subjective experiences, perceptions, and meaning-making in real OHCA events, thereby illuminating issues that are difficult to capture using quantitative indicators. Relevant qualitative studies have been conducted across different countries, cultural contexts, and emergency care systems, with variations in participants, settings, methods, and areas of emphasis. Consequently, the findings from individual studies cannot be readily translated into evidence of broad practical relevance. A systematic search, critical appraisal, and rigorous synthesis of primary qualitative research in this field is required.

This review used the Joanna Briggs Institute (JBI) meta-aggregation^[8]^ to systematically synthesize the experiences and perceptions of lay bystanders who witnessed and/or participated in real OHCA events, with the aim of informing improvements in bystander CPR training, emergency response systems, and related support measures.

## 2. Review Objective and Question

This review aimed to synthesize qualitative evidence regarding lay bystanders’ experiences and perceptions of responding to OHCA events.

Review question: What are the experiences and perceptions of lay bystanders who witness and/or participate in actual OHCA events, including the recognition of the event, the decision to intervene, CPR performance or AED use, and psychological adjustment after the event?

## 3. Methods

This review was conducted in accordance with the JBI methodology for qualitative systematic reviews^[8]^ and reported following the Preferred Reporting Items for Systematic Reviews and Meta-Analyses (PRISMA) 2020 statement,^[9]^ PRISMA for searching (PRISMA-S),^[10]^ and enhancing transparency in reporting the synthesis of qualitative research.^[11]^ This study was approved by the Ethics Committee of the Second Affiliated Hospital of Army Medical University (People’s Liberation Army, PLA) (approval no. 2025-Research-248-01).

### 3.1. Inclusion criteria

The following inclusion criteria were defined using the population, phenomenon of interest, and context framework for qualitative evidence synthesis

#### 3.1.1. Population

Lay bystanders who had witnessed an OHCA were included in this study. Participants who reported that they had professional healthcare capacities were excluded from this study. When a study used the term “first responder,” it was included only if the role was nonprofessional or if findings representing lay rescuers could be clearly extracted.

#### 3.1.2. Phenomenon of interest

The phenomenon of interest was the experience and perception of responding to OHCA, including event recognition, situational appraisal, motivators and inhibitors of action, CPR, interaction and teamwork, acute emotional responses, and post-event adjustment.

#### 3.1.3. Context

Eligible events occurred outside hospitals, including homes, workplaces, public locations, and community settings. Studies based solely on simulated scenarios and those on in-hospital cardiac arrests were excluded.

#### 3.1.4. Types of studies

Primary studies with qualitative designs including phenomenography, phenomenology, grounded theory, qualitative descriptions, and qualitative content analysis were eligible. Mixed-methods studies or those that primarily used quantitative methods were included only when they contained an independently identifiable qualitative component from which author-level findings could be extracted. Systematic reviews, editorials, guidelines, and quantitative studies were also excluded. Studies published in English or Chinese were included.

### 3.2. Search strategy

Controlled vocabulary and free-text terms were combined to develop search strategies to address cardiac arrest/OHCA, responses related to CPR, lay bystanders or 1st responders, experiences and perceptions, and qualitative research. PubMed, Embase, Cochrane Library, China National Knowledge Infrastructure, Wanfang Data, Chinese Scientific and Technological Journal Database, and Chinese Biomedical Literature Database were searched from database inception to July 31, 2026. An additional study was conducted using citation tracking. Appendix I, Supplemental Digital Content 1 provides the complete search strings, platforms, and search dates and yields for each database.

### 3.3. Study selection

The retrieved records were imported into Zotero 9.0 (Corporation for Digital Scholarship, https://www.zotero.org/) and de-duplicated. Two reviewers (SY and XJ) independently screened titles and abstracts against the eligibility criteria and assessed the full text of potentially relevant studies. Disagreements were resolved through discussion, and unresolved disagreements were adjudicated by a 3rd reviewer (SS).

### 3.4. Methodological quality appraisal

Two reviewers (SY and XJ) independently appraised potentially eligible studies by using the JBI critical appraisal checklist for qualitative research.^[8]^ The checklist comprises ten items that assess congruity from a philosophical perspective, research methodology, research question, data collection, data analysis, interpretation of findings, representation of participants’ voices, ethical conduct, and conclusions. Each item was rated as “yes, no, unclear, or not applicable.” A “yes” response was assigned 1 point, whereas “no, unclear, and not applicable” were assigned 0 points, and the total score was calculated. Disagreements were resolved through discussion, and unresolved disagreements were adjudicated by a 3rd reviewer (SS). Appraisal scores informed the interpretation of the evidence and confidence in the evidence from reviews of qualitative research (ConQual) grading but were not used as a threshold for exclusion.

### 3.5. Data extraction

Using a standardized extraction form, 2 reviewers (SY and XJ) independently extracted information regarding the study design, participants, context, phenomenon of interest, data collection, and data analysis. Disagreements were resolved through discussion, and unresolved disagreements were adjudicated by a 3rd reviewer (SS).

The credibility of each finding was determined from the congruence between the findings and supporting illustration. Findings with a clear association beyond reasonable doubt were rated unequivocal (U), those with a plausible but potentially contestable association were rated credible (C), and those not supported by the available data were rated unsupported. Only findings rated as U or C were included in the meta-analysis. The findings from the Chinese-language study were translated into English by the reviewers.

### 3.6. Data synthesis

Meta-aggregation proceeds on 3 levels. First, we extracted the authors’ original findings and accompanying illustrations. Second, the findings with sufficiently similar meanings were grouped into categories. Finally, the categories were aggregated into practically meaningful synthesized findings (SFs), while preserving the intent of the original authors.^[12]^

### 3.7. Assessment of confidence in the SFs

Confidence in the evidence for each SF was assessed using ConQual^[13]^ and presented in a summary of findings table. Because all included evidence was qualitative, each SF began with a high confidence rating. Dependability was judged using items 2, 3, 4, 6, and 7 of the JBI appraisal checklist: no downgrade was applied when 4 or 5 items were rated “yes,” 1 level was deducted when 2 or 3 items were rated “yes,” and 2 levels were deducted when 0 or 1 item was rated “yes.” Credibility was determined by the composition of U- and C-rated findings; no downgrade was applied when only U-rated findings were included, and 1 level was deducted when both U- and C-rated findings were included. Final confidence was rated as high, moderate, low, or very low according to the cumulative downgrading for dependability and credibility.

## 4. Results

### 4.1. Study selection

A database search yielded 478 records. After the removal of 85 duplicates, 393 records remained. Title and abstract screening excluded 359 records, leaving 34 reports for which the full text was sought. Five full-text reports could not be obtained because the authors’ institutions did not subscribe to relevant journals or databases and 29 full-text reports were assessed for eligibility. Twenty-two participants were excluded as they were not lay bystanders. Seven studies were identified through database searching, and 1 study was identified through citation tracking, yielding a total of 8 studies.^[14–21]^ The selection process is illustrated in the PRISMA 2020 flow diagram.

### 4.2. Characteristics of included studies

Eight studies were published between 2000 and 2024 and were conducted in Sweden, Germany (2 studies), Denmark, Norway, Canada, and China (2 studies), representing 6 countries.^[14–21]^ The qualitative components included 209 participants with sample sizes ranging from 9 to 89. The study design included phenomenology, grounded theory, exploratory qualitative research, and qualitative content analysis. Events occurred at homes, workplaces, public locations, community AED, and emergency response settings. Data were collected through face-to-face and telephone interviews and focus groups. The characteristics of the included studies are summarized in Table 1.

**Table 1:** Characteristics of included studies (N = 8).

Author (year), country	Study design	Participants (sample = n)	Context/setting	Phenomenon of interest	Data collection	Data analysis
Axelsson et al.^[14]^ (2000), Sweden	Qualitative study	Bystanders who performed CPR during an OHCA (n = 19)	Sweden; out-of-hospital cardiac arrest events involving lay bystanders	To describe how bystanders perceived their CPR intervention.	Individual, tape-recorded interviews guided by a question template/interview guide.	Phenomenographic analysis; findings organized into 5 main categories and 14 subcategories.
Beck et al.^[15]^ (2024), Germany	Qualitative interview study	Lay witnesses of at-home cardiac arrest (n = 21)	Berlin, Germany; cardiac arrests in patients’ homes	To explore factors facilitating and impeding B-CPR in at-home cardiac arrest and derive overarching concepts.	Face-to-face semi-structured interviews at the participant’s home or a quiet hospital room	Qualitative content analysis according to Mayring; category system, inductive derivation of 6 concepts.
Brinkrolf et al.^[16]^ (2021), Germany	Post hoc analysis of a semi-standardized telephone-interview dataset	Adult-OHCA witnesses (n = 89)	Münster, Germany; witnessed adult OHCA from December 2014 to April 2016	To examine bystanders’ psychological processing after OHCA and its associations with performing CPR and with relationship to the patient.	A semi-standardized telephone questionnaire included an open question about the paramount emotion; responses were recorded verbatim.	Two independent raters categorized responses.
Chen et al.^[17]^ (2020), Taiwan,China	Grounded theory	Lay rescuers from 5 events (n = 9)	Taiwan; public locations and the national Public AED registry/PAD program	To characterize experiences surrounding bystander-initiated resuscitation and psychological influences after the event in an Asian community.	Individual, in-depth, face-to-face semi-structured interviews using an 18-question outline; audio-recorded.	Content analysis of verbatim transcripts; coding into categories and subcategories. Two researchers analyzed independently and reached consensus.
Malta Hansen et al.^[18]^ (2017), Denmark	Qualitative study	128 lay bystanders were interviewed. Twenty-six recorded interviews were purposively selected for in-depth qualitative analysis (n = 26)	Denmark; authentic OHCAs involving a donated-AED network	To identify factors facilitating lay bystander CPR and AED use in authentic OHCA.	Semi-structured telephone interviews; recruitment/selection continued to data saturation.	Five-stage iterative, inductive cross-sectional indexing.
Mathiesen et al.^[19]^ (2016), Norway	Exploratory qualitative study	Lay rescuers from 18 OHCA events (n = 20)	Stavanger region, Norway; event-to-interview interval 6 days–13 years	To explore reactions and coping strategies among lay rescuers after providing CPR to OHCA victims.	18 face-to-face and 2 telephone in-depth semi-structured interviews; audio-ecorded.	Inductive qualitative content analysis (*Graneheim and Lundman*): meaning units, codes, subcategories, 3 categories and 1 overarching theme.
Mausz et al.^[20]^ (2018), Canada	Constructivist grounded theory (*Charmaz*)	Lay rescuers who had recently intervened in an OHCA (n = 15)	Ontario, Canada; participants recruited through Peel Regional Paramedic Services following recent OHCA interventions.	To explore bystander CPR and develop a mid-range explanatory theory of the lay-rescuer process.	One individual interview and 5 focus groups (2–7 participants), semi-structured, 45–90 minutes.	Constant comparative analysis with theoretical sampling until theoretical saturation; development of a 3-stage explanatory model.
Yang Lu et al.^[21]^ (2022), China	Phenomenological qualitative study	Bystanders with experience of performing CPR (n = 10)	Hainan, China; 1-to-1 interviews conducted in an independent, quiet setting.	To explore 1st responders’ experiences of performing CPR.	One-to-one semi-structured in-depth interviews (about 40 minutes), audio-recorded with field notes.	Colaizzi 7-step phenomenological analysis using NVivo 11.

AED = automated external defibrillator, B-CPR = bystander cardiopulmonary resuscitation, CPR = cardiopulmonary resuscitation, EMS = emergency medical services, OHCA = out-of-hospital cardiac arrest, PAD = public access defibrillation.

### 4.3. Methodological quality

Eight studies were included in this meta-analysis. Overall, there was good congruity between the research questions and the data collection, analysis, and interpretation. The most common limitation was the inadequate reporting of researchers’ cultural or theoretical positioning and reflexivity. The detailed appraisal results are presented in Table 2. Of the 5 JBI items used to assess ConQual dependability, the studies by Axelsson, Beck, Brinkrolf, and Yang each received 3 “yes” ratings,^[14–16,21]^ those by Chen and Mathiesen each received 4,^[17,19]^ and those by Malta Hansen and Mausz each received 5.^[18,20]^ The Brinkrolf study did not present corresponding supporting illustrations in the main report,^[16]^ and the supplementary appendix cited by the original authors could not be obtained. Consequently, the congruence between these 3 findings and their underlying data could not be established beyond reasonable doubt, and the findings were rated as C rather than U.

**Table 2:** Results of study quality assessment.

Study	(1)	(2)	(3)	(4)	(5)	(6)	(7)	(8)	(9)	(10)	Score
Axelsson et al.^[14]^ (2000)	Y	Y	Y	Y	Y	N	U	Y	Y	Y	8/10
Beck et al.^[15]^ (2024)	Y	Y	Y	Y	Y	N	N	Y	Y	Y	8/10
Brinkrolf et al.^[16]^ (2021)	N	Y	Y	Y	Y	N	N	Y	Y	Y	7/10
Chen et al.^[17]^ (2020)	Y	Y	Y	Y	Y	Y	U	Y	Y	Y	9/10
Malta Hansen et al.^[18]^ (2017)	U	Y	Y	Y	Y	Y	Y	Y	Y	Y	9/10
Mathiesen et al.^[19]^ (2016)	U	Y	Y	Y	Y	Y	U	Y	Y	Y	8/10
Mausz et al.^[20]^ (2018)	Y	Y	Y	Y	Y	Y	Y	Y	Y	Y	10/10
Yang Lu et al.^[21]^ (2022)	U	Y	Y	Y	Y	N	U	Y	Y	Y	7/10

(1) Is there congruity between the stated philosophical perspective and the research methodology?

(2) Is there congruity between the research methodology and the research question or objectives?

(3) Is there congruity between the research methodology and the methods used to collect data?

(4) Is there congruity between the research methodology and the representation and analysis of data?

(5) Is there congruity between the research methodology and the interpretation of results?

(6) Is there a statement locating the researcher culturally or theoretically?

(7) Is the influence of the researcher on the research, and vice versa, addressed?

(8) Are participants, and their voices, adequately represented?

(9) Is the research ethical according to current criteria or, for recent studies, is there evidence of ethical approval by an appropriate body?

(10) Do the conclusions drawn in the research report flow from the analysis, or interpretation, of the data?

N = no, U = unclear, Y = yes.

### 4.4. Meta-aggregation

Thirty-three findings were extracted from the original studies: 30 were rated U, 3 were C , and none were unsupported. These findings were grouped into 12 categories and then aggregated into 4 SFs (Table 3 and Appendix II, Supplemental Digital Content 2).

**Table 3:** Meta-aggregation of synthesized findings, categories, and original-author findings with level of credibility.

Synthesized finding	Category	Study findings
SF1: Bystander action is motivated by prosocial responsibility and automatic helping, but may be inhibited by perceived risks, repugnance, and psychological burden.	Category 1.1: Prosocial motivation, responsibility, courage, and automatic action	A1 (U): To have a sense of humanity (to wish to save lives; to wish to help; to act intuitively). A3 (U): To feel an obligation (to take responsibility; to do one’s duty). A4 (U): To have courage (to dare; to have an intrinsic power). B6 (U): Automaticity of actions. C1 (U): Motivation: The rescuers showed helping traits, individual cognition, and situation awareness, which were factors motivating the resuscitation efforts. Y1 (U): First responders had a strong sense of responsibility and empathy.
	Category 1.2: Exposure, repugnance, psychological burden, and perceived risk	A5 (U): To feel exposed (to feel deserted; powerless; ambivalent; uncertain; and repugnance). Y4 (U): First responders experienced a heavy psychological burden during CPR and found it difficult to regulate.
SF2: Unexpected OHCA presentations and misinterpreted signs delay recognition of cardiac arrest and the perceived need for immediate CPR.	Category 2.1: Unexpectedness and difficulty in situational judgement	B1 (U): Unexpectedness of the event. B3 (U): Situational judgement.
	Category 2.2: Misinterpretation of signs and failure to recognize the need for CPR	B4 (U): Awareness of the necessity to perform B-CPR. MA2 (U): Taking action to save the victim is complicated by misconceptions about cardiac arrest and by practical CPR/AED challenges.
SF3: Hands-on training, workable conditions, usable equipment, teamwork, and leadership facilitate resuscitation; limited preparedness and the training–reality gap impede performance.	Category 3.1: Preparedness, prior training, and the training–reality gap	A2 (U): To have competence (to have knowledge; to feel prepared). C2 (U): Training–reality discrepancies: Rescuers reported discrepancies between actual resuscitation, prior training experiences, and their expectations. H1 (U): Knowledge and skills from CPR courses: Previous hands-on CPR training and knowledge that intervention is vital and cannot cause substantial harm facilitated action. Y2 (U): First responders’ 1st-aid skills and competence needed improvement. Y3 (U): First responders lacked professional 1st-aid knowledge and literacy.
	Category 3.2: Physical, environmental, equipment, and technical conditions	B5 (U): Initial position of the patient. H2 (U): Ventilation masks: A mask helped bystanders overcome oral fluids and repugnance during ventilation. H3 (U): The AED apparatus: Familiarity with the AED, prior knowledge that it would guide CPR, and its voice prompts increased reassurance and confidence.
	Category 3.3: Teamwork and on-scene leadership	H4 (U): Bystander teamwork: Division and delegation of tasks helped the resuscitation attempt run effectively. H5 (U): Leadership and the moral imperative to act: Showing leadership and feeling a moral obligation drove participation.
SF4: Responding to OHCA may cause acute or persistent distress; recovery is shaped by psychological processing, patient outcomes, information, support, and meaning-making.	Category 4.1: Acute stress, panic, and shock	B2 (U): Acute stress response. MA1 (U): Being called to act is disturbing, causing panic, shock, and disbelief that must ultimately be overcome.
	Category 4.2: Persistent distress, self-blame, and difficulty making sense of the event	BR1 (C): Signs of pathological psychological processing. M1 (U): Concern. MA3 (U): Making sense of the experience is challenging: lay rescuers contend with self-doubt, unanswered questions, and uncomfortable emotional reactions.
	Category 4.3: Physiological psychological processing and limited or no distress	BR2 (C): Physiological psychological processing. BR3 (C): No signs of psychological distress due to the OHCA.
	Category 4.4: Outcome-dependent adjustment, recovery, and personal growth	C3 (U): Psychological influence: Reflection on the event involved mental strengthening, greater responsibility and confidence; short-term negative reactions also occurred, and rescuers remained willing to help in future. Y5 (U): CPR outcomes had a profound impact on 1st responders’ quality of life.
	Category 4.5: Coping, support, and access to patient-outcome information	M2 (U): Uncertainty. M3 (U): Coping strategies.

Only U and C findings are included in the meta-aggregation.

AED = automated external defibrillator, B-CPR = bystander cardiopulmonary resuscitation, C = credible, CPR = cardiopulmonary resuscitation, NS = not supported, OHCA = out-of-hospital cardiac arrest, SF = synthesized finding, U = unequivocal.

#### 4.4.1. Synthesized finding 1: motivators and inhibitors of bystander response

Social responsibility and an automatic helping response can prompt bystanders to act, whereas perceived risk, disgust, bodily exposure, and psychological burden can inhibit intervention. This finding was supported by 8 U findings from 4 studies.^[14,15,17,21]^

Category 1.1: Social motivation, responsibility, courage, and automatic action.

This category comprised 6 U findings from 4 studies: Axelsson et al, Beck et al, Chen et al, and Yang et al.^[14,15,17,21]^ Participants described responding as arising from humanitarian concern, empathy, responsibility, and obligation, or as an intuitive or automatic action that did not involve repeated deliberation. A prior sense of competence and situational awareness further strengthened their willingness to assume responsibility for their response.

Category 1.2: Exposure, disgust, psychological burden, and perceived risk.

This category comprises 2 U findings from the studies by Axelsson et al and Yang et al.^[14,21]^ Rescuers may feel isolated, powerless, conflicted, or uncertain. Contact with bodily fluids, infection risk, bodily exposure, or fear of causing harm can evoke disgust and hesitation, whereas an unmanageable psychological burden increases the pressure to continue resuscitating.

#### 4.4.2. Synthesized finding 2: recognition and appraisal of OHCA

An unexpected presentation of OHCA or misinterpretation of its signs could delay the recognition of cardiac arrest and appraisal of the need for immediate CPR. Therefore, recognition was not a single factual judgment, but a rapidly evolving interpretive process undertaken in a state of shock. Misconceptions and uncertainty regarding practical actions reinforce 1 another. This finding was supported by 4 U findings from 2 studies.^[15,20]^

Category 2.1: Unexpectedness of the event and difficulty with situational appraisal.

This category comprises 2 U findings from the study by Beck et al.^[15]^ Because the events occurred suddenly amid ordinary household activities, and family members found them difficult to interpret.

Category 2.2: Misinterpretation of signs and failure to recognize the need for CPR.

This category comprised 2 U findings from studies by Beck et al and Mausz et al.^[15,20]^ Abnormal breathing, an appearance of resting, or brief residual movement, could be misinterpreted as evidence that cardiac arrest had not occurred. Misconceptions about cardiac arrest and CPR/AED use also prevented some bystanders from recognizing the need for immediate CPR.

#### 4.4.3. Synthesized finding 3: conditions that facilitate or impede resuscitation performance

Hands-on training, workable on-scene conditions, available equipment, teamwork, and on-scene leadership facilitate resuscitation, whereas inadequate preparedness and the gaps between training and actual events impede performance. This finding was supported by 10 U findings from 5 studies.^[14,15,17,18,21]^

Category 3.1: Preparedness, previous training, and the training–reality gap.

This category comprised 5 U findings from 4 studies (Axelsson et al, Chen et al, Malta Hansen et al, and Yang et al).^[14,17,18,21]^ Recent or hands-on training provided bystanders with a script for action, which reinforced the belief that attempting CPR was preferable to doing nothing. Nevertheless, the participants also reported that their minds went blank, their knowledge was incomplete, they were uncertain about the compression force, and the mannequin training differed from that of a real patient’s body, appearance, movement, and emotional intensity. These discrepancies diminished the perceived competence at the scene.

Category 3.2: Physical, environmental, equipment-related, and technical conditions.

This category comprises 3 U findings from the studies by Beck et al and Malta Hansen et al.^[15,18]^ Environmental and technical conditions shaped the actions that could be performed at the scene. The patient’s initial position, body weight, confined spaces, blood or oral secretions, and difficulty in movement can impede resuscitation. A ventilation mask could reduce the disgust associated with bodily fluids, while familiarity with an AED and its voice prompts increases reassurance and confidence in its use.

Category 3.3: Teamwork and on-scene leadership.

This category comprises 2 U findings from the study by Hansen et al.^[18]^ Resuscitation is easier to sustain when bystanders divide tasks, maintain communication with the dispatcher, and accept on-scene leadership. Therefore, competence during a real event does not reside solely in an individual’s recall of CPR steps; it is distributed across the individual, environment, equipment, and response team. Clear task allocation, delegation, and on-scene leadership helped maintain the resuscitation process, while a leader’s sense of moral responsibility motivated the organization of others and continued involvement in the response.

#### 4.4.4. Synthesized finding 4: emotional impact and post-event adjustment

Participation in OHCA response can lead to acute or persistent distress. Post-event adjustment is shaped by psychological processing, patient outcomes, information, support, and meaning-making. This SF was supported by 11 findings (8 U and 3 C) from 6 studies.^[14,15,17,19–21]^

Category 4.1: Acute stress, panic, and shock.

This category comprised 2 U findings from studies by Beck et al and Mausz et al.^[15,20]^ When asked to respond or when directly confronted with the patient, bystanders may experience panic, shock, altered breathing, disbelief, and marked physiological arousal. They had to regain their sense of control within a short period of time before they could act.

Category 4.2: Persistent distress, self-blame, and difficulty making sense of the event.

This category comprised 3 findings (2 U and 1 C) from studies by Brinkrolf et al, Mathiesen et al, and Mausz et al.^[16,19,20]^ After the event, some rescuers experienced intrusive memories, nightmares, avoidance, self-blame, worry, and doubt. They repeatedly questioned whether they had acted correctly and whether a different response might have changed the outcome, making it difficult to understand and integrate the experience.

Category 4.3: Normal psychological processing and limited or no persistent distress.

This category comprises 2 C findings from the study by Brinkrolf et al.^[16]^ Some participants’ reactions reflected normal psychological processing, whereas others showed little or no apparent OHCA-related distress. These findings indicate heterogeneous psychological trajectories after resuscitation and suggest that persistent pathological responses do not occur in all rescuers.

Category 4.4: Outcome-related adjustment, recovery, and personal growth.

This category comprised 2 U findings from studies by Chen et al and Yang et al.^[17,21]^ Patient outcome can profoundly affect rescuers’ quality of life and post-event adjustment. Simultaneously, reflection on the event could foster psychological growth, a stronger sense of responsibility, and greater self-confidence, thereby sustaining a willingness to help again in the future.

Category 4.5: Coping, support, and access to information about patient outcome.

This category comprised 2 U findings from a study by Mathiesen et al.^[19]^ Patient outcome and access to information were important but not simple determinants of adjustment; an unknown outcome or death could intensify guilt and rumination, whereas feedback and recognition supported meaning-making. Bystanders commonly coped by talking to family members, colleagues, or people with healthcare knowledge, although some of them required professional assistance. These forms of support are important in helping rescuers process their experiences and resume their daily lives.

### 4.5. ConQual summary of findings

Confidence in SF1, SF2, and SF3 was rated as moderate. Dependability was downgraded by 1 level because the contributing studies were limited in terms of researcher positioning and reflexivity. Credibility was not downgraded because all the contributing findings were U. Confidence in SF4 was rated low: dependability was downgraded by 1 level, and credibility was downgraded by a further level because the finding included 8 U and 3 C findings. Details are presented in Table 4.

**Table 4:** Summary of findings (ConQual).

Systematic review title	Lay bystanders’ experiences and perceptions of responding to out-of-hospital cardiac arrest: a qualitative systematic review				
Population	Lay bystanders and 1st responders who witnessed an OHCA, including those who initiated, attempted, or considered CPR and/or AED use.				
Interest of phenomenon	Experiences and perceptions surrounding recognition of OHCA, decisions to act, real-world resuscitation, and post-event psychological impact.				
Context	OHCA events in community, public, workplace, and home settings across Sweden, Germany, Taiwan, Denmark, Norway, Canada, and China.				
Synthesized finding	Type of research	Dependability	Credibility	ConQual level	Comments
SF1: Bystander action is motivated by prosocial responsibility and automatic helping, but may be inhibited by perceived risks, repugnance, and psychological burden.	Qualitative	Downgrade 1 level	No change	Moderate	Dependability: Downgraded 1 level due to methodological limitations across the included primary studies. Credibility: No change; 8 U, 0 C.
SF2: Unexpected OHCA presentations and misinterpreted signs delay recognition of cardiac arrest and the perceived need for immediate CPR.	Qualitative	Downgrade 1 level	No change	Moderate	Dependability: Downgraded 1 level due to methodological limitations across the included primary studies. Credibility: No change; 4 U, 0 C.
SF3: Hands-on training, workable conditions, usable equipment, teamwork, and leadership facilitate resuscitation; limited preparedness and the training–reality gap impede performance.	Qualitative	Downgrade 1 level	No change	Moderate	Dependability: Downgraded 1 level due to methodological limitations across the included primary studies. Credibility: No change; 10 U, 0 C.
SF4: Responding to OHCA may cause acute or persistent distress; recovery is shaped by psychological processing, patient outcomes, information, support, and meaning-making.	Qualitative	Downgrade 1 level	Downgrade 1 level	Low	Dependability: Downgraded 1 level due to methodological limitations across the included primary studies. Credibility: Downgraded 1 level due to a mix of unequivocal (U) and credible (C) findings; 8 U and 3 C.

Only U and C findings are included in the meta-aggregation.

AED = automated external defibrillator, C = credible, CPR = cardiopulmonary resuscitation, NS = not supported, OHCA = out-of-hospital cardiac arrest, SF = synthesized finding, U = unequivocal.

## 5. Discussion

### 5.1. Principal interpretation

This review reframes lay responses to OHCA as a continuum, rather than as a single decision. Action begins with an encounter that carries a strong moral impetus but considerable cognitive ambiguity. Bystanders may act because helping feels automatic, obligatory, or humanitarian. However, the same person may simultaneously experience fear, disgust, exposure, and uncertainty. Once an action begins, performance depends not only on prior knowledge but also on whether the patient can be positioned appropriately, the equipment is understandable, assistance is available, and someone coordinates the scene. The event may continue psychologically long after professional responders have departed.

This process-oriented interpretation is consistent with broader evidence that confidence, perceived competence, emotional arousal, and situational appraisal influence the initiation of CPR.^[5,6,22]^ However, a recent qualitative meta-synthesis examined public willingness to participate in out-of-hospital CPR,^[23]^ the present review demonstrates how these factors are experienced across the phases before, during, and after intervention in real OHCA events. Similarly, a scientific statement on the experiences of lay rescuers called for emergency systems to view training, on-scene responses, and residual impacts as interrelated phases.^[4]^

### 5.2. Recognition should be trained under ambiguous conditions

Public messaging often depicts cardiac arrest as a clearly recognizable sudden collapse. Synthesized evidence indicates that bystanders may encounter abnormal breathing, apparent residual movement, awkward body position, or domestic contexts that interfere with appraisal. Current basic life support guidelines emphasize unresponsiveness and abnormal breathing; however, training must also help the public tolerate uncertainty and understand that agonal or intermittent breathing does not indicate recovery.^[2]^ Scenario-based education should therefore include ambiguous presentations and explicitly reassure the public that chest compressions can be started immediately when cardiac arrest is suspected.^[24–26]^

### 5.3. Training should reproduce the tasks of real resuscitation

CPR training has been identified as a key independent predictor of bystanders’ ability to promptly deliver 1st-aid interventions for OHCA.^[27,28]^ The gap between training and actual events extends beyond forgetting procedural steps. Responders must also manage the patients’ body weight, floor position, rib movement, body fluids, fatigue, social hesitation, and multiple simultaneous tasks. Training should integrate realistic task transitions: recognizing abnormal breathing, moving the patient when feasible, calling emergency services on speakerphones, allocating tasks, retrieving and attaching an AED, rotating compressors, and continuing despite unpleasant sensory stimuli. Brief refresher training remains valuable, but scenario fidelity, role clarity, and teamwork practice may be as important as recall of factual knowledge.

AED design and public education should emphasize that voice prompts can transfer a part of the rescuer’s cognitive burden to the device. Dispatcher guidance can likewise reduce uncertainty by describing what the bystander may observe, confirming the appropriateness of immediate action, and providing concise instructions that support on-scene leadership.

### 5.4. Post-event care is part of the emergency response system

The emotional consequences of OHCA vary between studies. Many bystanders adjust without experiencing severe or persistent distress, whereas others experience intrusive memories, guilt, sleep disturbances, avoidance, or a need for professional support. This variation indicates that post-event effects should not be ignored or universally pathologized. A stepped support pathway can include early acknowledgment, plain language information about common reactions, a mechanism for requesting an event review, communication of patient outcomes where ethically permissible, and screening or referral when distress persists. Previous research has identified uncertainty about the outcome and lack of feedback as important concerns for lay rescuers, whereas organized debriefing has been found to be valuable.^[29–31]^

Information sharing must comply with patient privacy requirements and local laws. Even when a specific outcome cannot be disclosed, relevant agencies can explain what usually occurs after a patient handover, acknowledge the value of the rescue attempt, correct misconceptions regarding responsibility, and provide contact details for support. Such communication may have reduced the sense that the bystander’s role ended abruptly when the ambulance departed.

### 5.5. Cultural and contextual considerations

The included studies spanned 6 countries for >2 decades. Responsibility, empathy, courage, and distress were evident across the settings. However, their expression may be shaped by training systems, dispatch practices, legal protection, family relationships, availability of AEDs, and cultural expectations of helping. Studies from mainland China and Taiwan have extended the evidence beyond Western settings; however, only 2 studies have represented the Asian context. Participants in the Danish study were trained bystanders in an AED volunteer network, and several studies recruited individuals who had already acted. Such samples may have underestimated the experiences of those who did not intervene, declined participation, or left the scene. Implementation should therefore be adapted locally and evaluated among populations with lower confidence, less training, or weaker links to formal emergency systems.

### 5.6. Potential role of digital health support

Although digital interventions were not directly evaluated in the included studies, the synthesized findings suggest that appropriately designed digital tools may help address uncertainty in OHCA recognition, limited access to real-time guidance, difficulties in locating AEDs, and unmet needs for post-event information and support. Telemedicine can provide health education, professional communication, and continuity of support across time and place, creating a technological basis for extending public emergency response systems across pre-event preparation, on-scene care, and post-event follow-ups.^[28]^ Mobile applications can deliver brief and repeated CPR/AED refresher training, present atypical OHCA scenarios, support psychological preparedness, and identify nearby AEDs. In this scene, they can integrate emergency calls, speakerphone dispatcher guidance, location services, and concise procedural prompts. After an event, secure digital platforms provide information on common psychological reactions, self-screening, routes for requesting an event review, and referral to professional support.

### 5.7. Limitations

This study has several limitations. First, the review protocol was currently registered and not publicly registered before the start of the study. To enhance transparency, the search, selection, appraisal, extraction, meta-aggregation, and ConQual grading processes have been reported. Second, the included studies varied in event setting, relationship between participants and patients, training experience, responder role, interval between the event and interview, and the depth of qualitative data. Third, the 3 findings from Brinkrolf et al were not accompanied by accessible supporting illustrations because the relevant supplementary appendix cited by the original authors could not be obtained; therefore, they were rated as C rather than U. Fourth, most participants had performed or attempted CPR, and this review may not adequately represent the experiences of those who did not. Finally, confidence was moderate for the 3 synthesized findings and low for the post-event synthesized finding. Therefore, the recommendations should be evaluated during implementation rather than interpreted as definitive causal conclusions.

## 6. Conclusions

Lay bystanders are not a passive link between patient collapse and professional care. They must interpret uncertain signs, assume moral and emotional responsibilities, perform physically and socially complex tasks, and continue to experience the effects of an event long after the patient is transported. The evidence supports an integrated, rescuer-centered pathway that provides realistic preparation before an event, clear cognitive and interpersonal support during the response, and information and psychological support proportionate to the need afterward. Such a pathway may make public intervention more feasible while also recognizing the well-being of those who step forward to help.

## Author contributions

**Investigation:** Mei Yang, Maodan Liu, Hanting Liu.

**Project administration:** Shisi Li.

**Writing – original draft:** Shuyu Zhan, Xiaojiao Jiang.




