# Supplementary material for: Experiences and perceptions of lay bystanders responding to out-of-hospital cardiac arrest: A qualitative systematic review
Source: Medicine (Baltimore). 2026 Sep 4;105(36):e50530. doi: 10.1097/MD.0000000000050530 (PMC13549513; doi:10.1097/MD.0000000000050530)
Supplement: Supplementary file 1 [file medi-105-e50530-s001.docx]

**Appendix I: Search strategy**

**PubMed**

*Platform: PubMed.gov*

*Last searched: 31 July 2026*

| **Search** | **Query** | **Records retrieved** |
| --- | --- | --- |
| #1 | ("cardiac arrest"[MeSH] OR "out-of-hospital cardiac arrest"[MeSH]) OR ("cardiac arrest"[tiab] OR "heart arrest"[tiab] OR asystole[tiab] OR "cardiopulmonary arrest"[tiab]) | 62,295 |
| #2 | ("Cardiopulmonary Resuscitation"[MeSH] OR "Basic Cardiac Life Support"[MeSH]) OR ("Cardiopulmonary Resuscitation"[tiab] OR "Cardio-Pulmonary Resuscitation"[tiab] OR CPR[tiab] OR "Mouth-to-Mouth Resuscitation"[tiab] OR "Basic Cardiac Life Support"[tiab] OR resuscitation[tiab]) | 94,990 |
| #3 | ("Bystander Effect"[MeSH] OR "First Aid"[MeSH]) OR (rescue[tiab] OR bystander[tiab] OR "first witness"[tiab] OR "first responder"[tiab] OR "first responders"[tiab] OR "lay person"[tiab] OR "lay persons"[tiab] OR "lay people"[tiab] OR volunteer[tiab] OR volunteers[tiab]) | 346,639 |
| #4 | ("Attitude to Health"[MeSH] OR "Cognition"[MeSH] OR "Emotions"[MeSH] OR "Stress, Psychological"[MeSH] OR "Adaptation, Psychological"[MeSH]) OR (Attitude[tiab] OR cognition[tiab] OR emotion[tiab] OR emotions[tiab] OR psychology[tiab] OR experience[tiab] OR experiences[tiab] OR perception[tiab] OR perceptions[tiab] OR feeling[tiab] OR feelings[tiab] OR perspective[tiab] OR perspectives[tiab] OR concern[tiab] OR concerns[tiab] OR fear[tiab] OR fears[tiab] OR anxiety[tiab] OR stress[tiab] OR coping[tiab] OR barrier[tiab] OR barriers[tiab] OR facilitator[tiab] OR facilitators[tiab]) | 5,481,209 |
| #5 | ("Qualitative Research"[MeSH] OR "Anthropology, Cultural"[MeSH] OR "Focus Groups"[MeSH]) OR (qualitative[tiab] OR interview[tiab] OR interviews[tiab] OR "focus group"[tiab] OR "focus groups"[tiab] OR phenomenology[tiab] OR phenomenological[tiab] OR "grounded theory"[tiab] OR ethnography[tiab] OR ethnographic[tiab] OR narrative[tiab] OR narratives[tiab] OR "thematic analysis"[tiab] OR "content analysis"[tiab] OR interpretive[tiab] OR exploratory[tiab] OR "mixed method"[tiab] OR "mixed methods"[tiab]) | 1,274,250 |
| #6 | (#1 AND #2 AND #3 AND #4 AND #5) | 99 |

**Cochrane Library**

*Platform:Cochrane Library (Wiley)*

*Last searched: 31 July 2026*

| **Search** | **Query** | **Records retrieved** |
| --- | --- | --- |
| #1 | "cardiac arrest" OR "heart arrest" OR asystole OR "cardiopulmonary arrest" | 7,549 |
| #2 | "Cardiopulmonary Resuscitation" OR "Cardio-Pulmonary Resuscitation" OR CPR OR "Mouth-to-Mouth Resuscitation" OR "Basic Cardiac Life Support" | 4,790 |
| #3 | rescue OR bystander OR "first witness" OR (first NEXT responder*) OR (lay NEXT person*) OR "lay people" OR volunteer* | 120,581 |
| #4 | Attitude OR cognition OR emotion OR psychology OR experience* OR perception* OR feeling* OR perspective* OR concern* OR fear* OR anxiety* OR stress* OR coping OR barrier* OR facilitator* | 527,373 |
| #5 | Ethnography OR phenomenology OR "grounded theory" OR interview* OR "qualitative research" OR "qualitative study" OR (mixed NEXT method*) OR (thematic NEXT analysis) OR (content NEXT analysis) OR (focus NEXT group*) OR narrative* OR exploratory | 111,989 |
| #6 | #1 AND #2 AND #3 AND #4 AND #5 | 119 |

**Embase**

*Platform:[Embase.com](https://embase.com/) (Elsevier)*

*Last searched: 31 July 2026*

| **Search** | **Query** | **Records retrieved** |
| --- | --- | --- |
| #1 | 'cardiac arrest':ab,ti OR 'heart arrest':ab,ti OR asystole:ab,ti OR 'cardiopulmonary arrest':ab,ti | 97,990 |
| #2 | 'cardiopulmonary resuscitation':ab,ti OR 'cardio-pulmonary resuscitation':ab,ti OR cpr:ab,ti OR 'mouth-to-mouth resuscitation':ab,ti OR 'basic cardiac life support':ab,ti OR resuscitation:ab,ti | 133,351 |
| #3 | rescue:ab,ti OR bystander:ab,ti OR 'first witness':ab,ti OR 'first responder':ab,ti OR 'first responders':ab,ti OR 'lay person':ab,ti OR 'lay persons':ab,ti OR 'lay people':ab,ti OR volunteer:ab,ti OR volunteers:ab,ti | 500,747 |
| #4 | attitude:ab,ti OR cognition:ab,ti OR emotion:ab,ti OR emotions:ab,ti OR psychology:ab,ti OR experience:ab,ti OR experiences:ab,ti OR perception:ab,ti OR perceptions:ab,ti OR feeling:ab,ti OR feelings:ab,ti OR perspective:ab,ti OR perspectives:ab,ti OR concern:ab,ti OR concerns:ab,ti OR fear:ab,ti OR fears:ab,ti OR anxiety:ab,ti OR stress:ab,ti OR coping:ab,ti OR barrier:ab,ti OR barriers:ab,ti OR facilitator:ab,ti OR facilitators:ab,ti | 6,156,730 |
| #5 | qualitative:ab,ti OR interview:ab,ti OR interviews:ab,ti OR 'focus group':ab,ti OR 'focus groups':ab,ti OR phenomenology:ab,ti OR phenomenological:ab,ti OR 'grounded theory':ab,ti OR ethnography:ab,ti OR ethnographic:ab,ti OR narrative:ab,ti OR narratives:ab,ti OR 'thematic analysis':ab,ti OR 'content analysis':ab,ti OR interpretive:ab,ti OR exploratory:ab,ti OR 'mixed method':ab,ti OR 'mixed methods':ab,ti | 1,390,139 |
| #6 | #1 AND #2 AND #3 AND #4 AND #5 | 138 |

**China National Knowledge Infrastructure (CNKI)**

*Platform::[cnki.net](https://cnki.net/)*

*Last searched: 31 July 2026*

| **Search** | **Query (Chinese)** | **Date Limit** | **Records retrieved** |
| --- | --- | --- | --- |
| #1 | SU % = ("cardiac arrest" OR "out-of-hospital cardiac arrest" OR "cardiopulmonary resuscitation" OR "CPR" OR "resuscitation") AND SU % = ("bystander" OR "witness" OR "rescuer" OR "public" OR "volunteer") AND SU % = ("experience" OR "feeling" OR "attitude" OR "cognition" OR "psychology" OR "perception" OR "coping") AND SU % = ("qualitative research" OR "qualitative study" OR "interview" OR "phenomenology" OR "grounded theory" OR "content analysis") | Inception–2025 | 11 |

*Note:English translation of the search strategy for the Chinese-language database.*

**Wanfang Data**

*Platform:wanfangdata.com.cn*

*Last searched: 31 July 2026*

| **Search** | **Query (Chinese)** | **Date Limit** | **Records retrieved** |
| --- | --- | --- | --- |
| #1 | SU % = ("cardiac arrest" OR "out-of-hospital cardiac arrest" OR "cardiopulmonary resuscitation" OR "CPR" OR "resuscitation") AND SU % = ("bystander" OR "witness" OR "rescuer" OR "public" OR "volunteer") AND SU % = ("experience" OR "feeling" OR "attitude" OR "cognition" OR "psychology" OR "perception" OR "coping") AND SU % = ("qualitative research" OR "qualitative study" OR "interview" OR "phenomenology" OR "grounded theory" OR "content analysis") | Inception–2025 | 47 |

*Note:English translation of the search strategy for the Chinese-language database.*

**Chinese Scientific and Technological Journal Database (CQVIP)**

*Platform:[cqvip.com](https://cqvip.com/)*

*Last searched: 31 July 2026*

| **Search** | **Query ( Chinese)** | **Records retrieved** |
| --- | --- | --- |
| #1 | (M="cardiac arrest" OR M="out-of-hospital cardiac arrest" OR M="cardiopulmonary resuscitation" OR M="CPR" OR M="resuscitation") AND (M="bystander" OR M="witness" OR M="rescuer" OR M="public" OR M="volunteer") AND (M="experience" OR M="feeling" OR M="attitude" OR M="cognition" OR M="psychology" OR M="perception" OR M="coping") AND (M="qualitative research" OR M="qualitative study" OR M="interview" OR M="phenomenology" OR M="grounded theory" OR M="content analysis") | 1 |

*Note:English translation of the search strategy for the Chinese-language database.*

**Chinese Biomedical Literature Database (CBM)**

*Platform:[sinomed.ac.cn](https://sinomed.ac.cn/)*

*Last searched: 31 July 2026*

| **Search** | **Query ( Chinese)** | **Records retrieved** |
| --- | --- | --- |
| #1 | ("experience" OR "feeling" OR "attitude") AND (cardiopulmonary resuscitation AND (public OR rescuer OR family members)) | 63 |

*Note:English translation of the search strategy for the Chinese-language database.*

**Abbreviations**

- OHCA：out-of-hospital cardiac arrest;
- CPR: cardiopulmonary resuscitation;
- AED: automated external defibrillator;
- CNKI:China National Knowledge Infrastructure
- CQVIP:Chinese Scientific and Technological Journal Database
- CBM: Chinese Biomedical Literature Database
