# Supplementary material for: Experiences and perceptions of lay bystanders responding to out-of-hospital cardiac arrest: A qualitative systematic review
Source: Medicine (Baltimore). 2026 Sep 4;105(36):e50530. doi: 10.1097/MD.0000000000050530 (PMC13549513; doi:10.1097/MD.0000000000050530)
Supplement: Supplementary file 2 [file medi-105-e50530-s002.docx]

**Experiences and Perceptions of Lay Bystanders Responding to Out-of-Hospital Cardiac Arrest: A Qualitative Systematic Review**

**Authors:** Shuyu Zhan , BS^1^,^*^; Xiaojiao Jiang , BS^1^,^*^; Mei Yang , BS^1^; Maodan Liu , BS^1^; Hanting Liu , BS ^1^; Shisi Li , BS^1^,^#^

**Affiliations:** ^1^ Department of Cardiovascular Surgery, The Second Affiliated Hospital Of Army Medical University: Xinqiao Hospital, Chongqing, 400037, China.

**Corresponding author:** ^#^ Shisi Li, BS: 286128283@qq.com,Tel: +86-13637823166.

**Explain:** ^*^These authors contributed equally to this work.

# Abbreviations

- JBI: Joanna Briggs Institute
- ConQual: Confidence in the Evidence from Reviews of Qualitative research
- OHCA：Out-of-Hospital Cardiac Arrest;
- CPR: Cardiopulmonary Resuscitation;
- AED: Automated External Defibrillator;
- PRISMA: Systematic Reviews and Meta-Analyses
- PRISMA-S: PRISMA for Searching
- ENTREQ: Enhancing Transparency in Reporting the Synthesis of Qualitative Research
- PICo:Population, phenomenon of Interest, and Context
- CNKI:China National Knowledge Infrastructure
- CQVIP:Chinese Scientific and Technological Journal Database
- CBM: Chinese Biomedical Literature Database

# Abstract

**Objective:** To synthesize the experiences and perceptions of lay bystanders before, during, and after responding to out-of-hospital cardiac arrests.

**Methods:** Seven English and Chinese databases were searched from their inception to July 31, 2026, supplemented with citation tracking. Two reviewers independently selected studies, appraised methodological quality using the Joanna Briggs Institute (JBI) Critical Appraisal Checklist for Qualitative Research, and extracted the findings and supporting illustrations. Unequivocal and credible findings were synthesized using JBI meta-aggregation, and confidence was assessed using Confidence in the Evidence from Reviews of Qualitative Research (ConQual).

**Results:** Eight studies involving 209 participants were included. Thirty-three findings were grouped into 12 categories and four were synthesized findings concerning the motivators and inhibitors of response, recognition and appraisal of out-of-hospital cardiac arrest, conditions facilitating or impeding cardiopulmonary resuscitation and automated external defibrillator use, and post-event psychological responses and adjustment. ConQual confidence was moderate for the first three synthesized findings, and low for the fourth.

**Conclusions:** Bystander response is a continuum that extends from event recognition and action to post-event adjustment. Out-of-hospital cardiac arrest systems should provide realistic preparations, clear on-scene support, proportionate information, and psychological support after an event.

**Keywords:** Out-of-Hospital Cardiac Arrest; Cardiopulmonary Resuscitation; Lay bystander; Qualitative systematic review

# 1. Introduction

Out-of-hospital cardiac arrest (OHCA), characterized by the abrupt cessation of cardiac mechanical activity and effective circulation outside the hospital, is a major public health emergency.^1^ OHCA is highly time-sensitive, and survival depends on early recognition, prompt activation of emergency medical services, high-quality cardiopulmonary resuscitation (CPR), and early use of an automated external defibrillator (AED).^2^ Because professional emergency responders are seldom able to reach the scene immediately, lay bystanders are often the first to activate a chain of survival. However, their presence does not necessarily translate into timely and effective action. A study conducted in Pakistan reported that a bystander was present in nearly 90% of OHCA events; however, only 2.3% of patients received bystander CPR,^3^ indicating a substantial gap between witnessing an event and intervening.

Previous OHCA research has primarily focused on the rates of bystander CPR and AED use, emergency response times, and patient survival, with comparatively limited attention paid to rescuer experiences. The American Heart Association has emphasized that bystanders are integral participants in OHCA systems of care, and that their training, involvement at the scene, and related experiences warrant systematic attention.^4^ Existing evidence suggests that the initiation of CPR depends not only on knowledge and skills but also on interacting psychological, behavioral, and situational factors.^5,6^ Much of this evidence is derived from quantitative or mixed-methods research and therefore cannot fully explain how bystanders interpret real events or make sense of their resuscitation experiences.

Qualitative research^7^ can provide in-depth accounts of participants' subjective experiences, perceptions, and meaning-making in real OHCA events, thereby illuminating issues that are difficult to capture using quantitative indicators. Relevant qualitative studies have been conducted across different countries, cultural contexts, and emergency care systems, with variations in participants, settings, methods, and areas of emphasis. Consequently, the findings from individual studies cannot be readily translated into evidence of broad practical relevance. A systematic search, critical appraisal, and rigorous synthesis of primary qualitative research in this field is required.

This review used the Joanna Briggs Institute (JBI) meta-aggregation^8^ to systematically synthesize the experiences and perceptions of lay bystanders who witnessed and/or participated in real OHCA events, with the aim of informing them of improvements in bystander CPR training, emergency response systems, and related support measures.

# 2. Review Objective and Question

This review aimed to synthesize qualitative evidence regarding lay bystanders' experiences and perceptions of responding to OHCA events.

Review question: What are the experiences and perceptions of lay bystanders who witness and/or participate in actual OHCA events, including the recognition of the event, decision to intervene, CPR performance or AED use, and psychological adjustment after the event?

# 3. Methods

This review was conducted in accordance with the JBI methodology for qualitative systematic reviews^8^ and reported following the Preferred Reporting Items for Systematic Reviews and Meta-Analyses (PRISMA) 2020 statement,^9^ (PRISMA for Searching) PRISMA-S,^10^ and Enhancing Transparency in Reporting the Synthesis of Qualitative Research (ENTREQ).^11^ This study was approved by the Ethics Committee of the Second Affiliated Hospital of Army Medical University (People's Liberation Army, PLA) (approval no. 2025-Research-248-01).

## 3.1 Inclusion Criteria

### The following inclusion criteria were defined using the population, phenomenon of Interest, and Context (PICo) framework for qualitative evidence synthesis.

### Population

Lay bystanders who had witnessed an OHCA were included in this study. Participants who responded that they had professional healthcare capacities were excluded from this study. When a study used the term 'first responder,' it was included only if the role was nonprofessional or if findings representing lay rescuers could be clearly extracted.

### Phenomenon of interest

The phenomenon of interest was the experience and perception of responding to OHCA, including event recognition, situational appraisal, motivators and inhibitors of action, CPR, interaction and teamwork, acute emotional responses, and post-event adjustment.

### Context

Eligible events occurred outside the hospitals, including homes, workplaces, public locations, and community settings. Studies based solely on simulated scenarios and those on in-hospital cardiac arrests were excluded.

### Types of studies

Primary studies with qualitative designs including phenomenography, phenomenology, grounded theory, qualitative descriptions, and qualitative content analysis were eligible. Mixed-methods studies or those that primarily used quantitative methods were included only when they contained an independently identifiable qualitative component from which author-level findings could be extracted. Systematic reviews, editorials, guidelines, and quantitative studies were also excluded. Studies published in English or Chinese were included.

## 3.2 Search Strategy

Controlled vocabulary and free-text terms were combined to develop search strategies to address cardiac arrest/OHCA, responses related to CPR, lay bystanders or first responders, experiences and perceptions, and qualitative research. PubMed, Embase, Cochrane Library, China National Knowledge Infrastructure (CNKI), Wanfang Data, Chinese Scientific and Technological Journal Database (CQVIP), and Chinese Biomedical Literature Database (CBM) were searched from database inception to July 31, 2026. An additional study has been conducted using citation tracking. *Appendix I* provides the complete search strings, platforms, and search dates and yields for each database.

## 3.3 Study Selection

The retrieved records were imported into Zotero 9.0 and de-duplicated. Two reviewers (SY and XJ) independently screened titles and abstracts against the eligibility criteria and assessed the full text of potentially relevant studies. Disagreements were resolved through discussion and unresolved disagreements were adjudicated by a third reviewer (SS).

## 3.4 Methodological Quality Appraisal

Two reviewers (SY and XJ) independently appraised potentially eligible studies by using the JBI Critical Appraisal Checklist for Qualitative Research.^8^ The checklist comprises ten items that assess congruity from a philosophical perspective, research methodology, research question, data collection, data analysis, interpretation of findings, representation of participants' voices, ethical conduct, and conclusions. Each item was rated as yes, no, unclear, ' or 'not applicable. ’ A 'yes' response was assigned one point, whereas’ no, unclear, and 'not applicable' were assigned zero points, and the total score was calculated. Disagreements were resolved through discussion and unresolved disagreements were adjudicated by a third reviewer (SS). Appraisal scores informed the interpretation of the evidence and Confidence in the Evidence from Reviews of Qualitative research (ConQual) grading but were not used as a threshold for exclusion.

## 3.5 Data Extraction

Using a standardized extraction form, two reviewers (SY and XJ) independently extracted information regarding the study design, participants, context, phenomenon of interest, data collection, and data analysis. Disagreements were resolved through discussion and unresolved disagreements were adjudicated by a third reviewer (SS).

The credibility of each finding was determined from the congruence between the findings and supporting illustration. Findings with a clear association beyond reasonable doubt were rated unequivocal (U), those with a plausible but potentially contestable association were rated credible (C), and those not supported by the available data were rated unsupported (NS). Only findings rated as U or C were included in the meta-analysis. The findings from the Chinese-language study were translated into English by the reviewers.

## 3.6 Data Synthesis

Meta-aggregation proceeds on three levels: First, we extracted the authors’ original findings and accompanying illustrations. Second, the findings with sufficiently similar meanings were grouped into categories. Finally, the categories were aggregated into practically meaningful synthesized findings, while preserving the intent of the original authors.^12^

## 3.7 Assessment of Confidence in the Synthesized Findings

Confidence in the evidence for each synthesized finding was assessed using ConQual ^13^ and presented in a Summary of Findings table. Because all included evidence was qualitative, each synthesized finding began with a high confidence rating. Dependability was judged using items 2, 3, 4, 6, and 7 of the JBI appraisal checklist: no downgrade was applied when four or five items were rated 'yes,' one level was deducted when two or three items were rated 'yes,' and two levels were deducted when zero or one item was rated 'yes.' Credibility was determined by the composition of U- and C-rated findings; no downgrade was applied when only U-rated findings were included and one level was deducted when both U- and C-rated findings were included. Final confidence was rated as high, moderate, low, or very low according to the cumulative downgrading for dependability and credibility.

# 4. Results

## 4.1 Study Selection

A database search yielded 478 records. After the removal of 85 duplicates, 393 records remained. Title and abstract screening excluded 359 records, leaving 34 reports for which the full text was sought. Five full-text reports could not be obtained because the authors' institutions did not subscribe to relevant journals or databases and 29 full-text reports were assessed for eligibility. Twenty-two participants were excluded as they were not lay bystanders. Seven studies were identified through database searching, and one study was identified through citation tracking, yielding a total of eight studies.^14–21^ The selection process is illustrated in *PRISMA 2020 flow diagram*.

## 4.2 Characteristics of Included Studies

Eight studies were published between 2000 and 2024 and were conducted in Sweden, Germany (two studies), Denmark, Norway, Canada, and China (two studies), representing six countries.^14–21^ The qualitative components included 209 participants with sample sizes ranging from 9 to 89. The study design included phenomenology, grounded theory, exploratory qualitative research, and a qualitative content analysis. Events occurred at homes, workplaces, public locations, community AED, and emergency response settings. Data were collected through face-to-face and telephone interviews and focus groups. The characteristics of the included studies are summarized in *Table 1.*

## 4.3 Methodological Quality

Eight studies were included in this meta-analysis. Overall, there was good congruity between the research questions and the data collection, analysis, and interpretation. The most common limitation was the inadequate reporting of researchers' cultural or theoretical positioning and reflexivity. The detailed appraisal results are presented in *Table 2.* Of the five JBI items used to assess ConQual dependability, the studies by Axelsson, Beck, Brinkrolf, and Yang each received three 'yes' ratings,^14–16,21^ those by Chen and Mathiesen each received four,^17,19^ and those by Malta Hansen and Mausz each received five.^18,20^ The Brinkrolf study did not present corresponding supporting illustrations in the main report,^16^ and the supplementary appendix cited by the original authors could not be obtained. Consequently, the congruence between these three findings and their underlying data could not be established beyond reasonable doubt, and the findings were rated as credible rather than unequivocal.

## 4.4 Meta-aggregation

Thirty-three findings were extracted from the original studies: 30 were rated unequivocal, three were credible, and none were unsupported. These findings were grouped into 12 categories and then aggregated into four synthesized findings (*Table 3 and Appendix II*).

### 4.4.1 Synthesized Finding 1: Motivators and Inhibitors of Bystander Response

Social responsibility and an automatic helping response can prompt bystanders to act, whereas perceived risk, disgust, bodily exposure, and psychological burden can inhibit intervention. This finding was supported by eight unequivocal findings from four studies.^14,15,17,21^

#### Category 1.1: Social motivation, responsibility, courage, and automatic action

This category comprised six unequivocal findings from four studies: Axelsson et al., Beck et al., Chen et al., and Yang et al..^14,15,17,21^ Participants described responding as arising from humanitarian concern, empathy, responsibility, and obligation, or as an intuitive or automatic action that did not involve repeated deliberation. A prior sense of competence and situational awareness further strengthened their willingness to assume responsibility for their response.

#### Category 1.2: Exposure, disgust, psychological burden, and perceived risk

This category comprises two unequivocal findings from the studies by Axelsson et al. and Yang et al.^14,21^. Rescuers may feel isolated, powerless, conflicted or uncertain. Contact with bodily fluids, infection risk, bodily exposure, or fear of causing harm can evoke disgust and hesitation, whereas an unmanageable psychological burden increases the pressure to continue resuscitating.

### 4.4.2 Synthesized Finding 2: Recognition and Appraisal of OHCA

An unexpected presentation of OHCA or misinterpretation of its signs could delay the recognition of cardiac arrest and appraisal of the need for immediate CPR. Therefore, recognition was not a single factual judgment, but a rapidly evolving interpretive process undertaken in a state of shock. Misconceptions and uncertainty regarding practical actions reinforce one another. This finding was supported by four unequivocal findings from two studies.^15,20^

#### Category 2.1: Unexpectedness of the event and difficulty with situational appraisal

This category comprises two unequivocal findings from the study by Beck et al..^15^ Because the events occurred suddenly amid ordinary household activities, and family members found them difficult to interpret.

#### Category 2.2: Misinterpretation of signs and failure to recognize the need for CPR

This category comprised two unequivocal findings from studies by Beck et al. and Mausz et al..^15,20^ Abnormal breathing, an appearance of resting or brief residual movement, could be misinterpreted as evidence that cardiac arrest had not occurred. Misconceptions about cardiac arrest and CPR/AED use also prevented some bystanders from recognizing the need for immediate CPR.

### 4.4.3 Synthesized Finding 3: Conditions That Facilitate or Impede Resuscitation Performance

Hands-on training, workable on-scene conditions, available equipment, teamwork, and on-scene leadership facilitate resuscitation, whereas inadequate preparedness and the gaps between training and actual events impede performance. This finding was supported by 10 unequivocal findings from five studies.^14,15,17,18,21^

#### Category 3.1: Preparedness, previous training, and the training-reality gap

This category comprised five unequivocal findings from four studies (Axelsson et al., Chen et al., Malta Hansen et al., and Yang et al.).^14,17,18,21^ Recent or hands-on training provided bystanders with a script for action, which reinforced the belief that attempting CPR was preferable to doing nothing. Nevertheless, the participants also reported that their minds went blank, their knowledge was incomplete, they were uncertain about the compression force, and the mannequin training differed from that of a real patient's body, appearance, movement, and emotional intensity. These discrepancies diminished the perceived competence in the scene.

#### Category 3.2: Physical, environmental, equipment-related, and technical conditions

This category comprises three unequivocal findings from the studies by Beck et al. and Malta Hansen et al..^15,18^ Environmental and technical conditions shaped the actions that could be performed at the scene. The patient's initial position, body weight, confined spaces, blood or oral secretions, and difficulty in movement can impede resuscitation. A ventilation mask could reduce the disgust associated with bodily fluids, while familiarity with an AED and its voice prompts increases reassurance and confidence in its use.

#### Category 3.3: Teamwork and on-scene leadership

This category comprises two unequivocal findings from the study by Hansen et al..^18^ Resuscitation is easier to sustain when bystanders divide tasks, maintain communication with the dispatcher, and accept on-scene leadership. Therefore, competence during a real event does not reside solely in an individual's recall of CPR steps; it is distributed across the individual, environment, equipment, and response team. Clear task allocation, delegation, and on-scene leadership helped maintain the resuscitation process, while a leader's sense of moral responsibility motivated the organization of others and continued involvement in the response.

### 4.4.4 Synthesized Finding 4: Emotional Impact and Post-event Adjustment

Participation in OHCA response can lead to acute or persistent distress. Post-event adjustment is shaped by psychological processing, patient outcomes, information, support, and meaning making. This synthesized finding was supported by 11 findings (eight unequivocal and three credible) from six studies.^14,15,17,19–21^

#### Category 4.1: Acute stress, panic, and shock

This category comprised two unequivocal findings from studies by Beck et al. and Mausz et al..^15,20^ When asked to respond or when directly confronted with the patient, bystanders may experience panic, shock, altered breathing, disbelief, and marked physiological arousal. They had to regain their sense of control within a short period of time before they could act.

#### Category 4.2: Persistent distress, self-blame, and difficulty making sense of the event

This category comprised three findings (two unequivocal and one credible) from studies by Brinkrolf et al., Mathiesen et al., and Mausz et al..^16,19,20^ After the event, some rescuers experienced intrusive memories, nightmares, avoidance, self-blame, worry, and doubt. They repeatedly questioned whether they had acted correctly and whether a different response might have changed the outcome, making it difficult to understand and integrate the experience.

#### Category 4.3: Normal psychological processing and limited or no persistent distress

This category comprises two credible findings from the study by Brinkrolf et al..^16^ Some participants' reactions reflected normal psychological processing, whereas others showed little or no apparent OHCA-related distress. These findings indicate heterogeneous psychological trajectories after resuscitation, and suggest that persistent pathological responses do not occur in all rescuers.

#### Category 4.4: Outcome-related adjustment, recovery, and personal growth

This category comprised two unequivocal findings from studies by Chen et al. and Yang et al..^17,21^ Patient outcome can profoundly affect rescuers' quality of life and post-event adjustment. Simultaneously, reflection on the event could foster psychological growth, a stronger sense of responsibility, and greater self-confidence, thereby sustaining a willingness to help again in the future.

#### Category 4.5: Coping, support, and access to information about patient outcome

This category comprised two unequivocal findings from a study by Mathiesen et al..^19^ Patient outcome and access to information were important but not simple determinants of adjustment; an unknown outcome or death could intensify guilt and rumination, whereas feedback and recognition supported meaning-making. Bystanders commonly coped by talking to family members, colleagues, or people with healthcare knowledge, although some of them required professional assistance. These forms of support are important in helping rescuers process their experiences and resume their daily lives.

## 4.5 ConQual Summary of Findings

Confidence in finding 1 (SF1), finding 2 (SF2), and finding 3 (SF3) was rated as moderate. Dependability was downgraded by one level because the contributing studies were limited in terms of researcher positioning and reflexivity. Credibility was not downgraded because all the contributing findings were unequivocal. Confidence in Synthesized Finding 4 (SF4) was rated low: dependability was downgraded by one level, and credibility was downgraded by a further level because the finding included eight unequivocal and three credible findings. Details are presented in *Table 4*.

# 5. Discussion

## 5.1 Principal Interpretation

This review reframes lay responses to OHCA as a continuum, rather than as a single decision. Action begins with an encounter that carries a strong moral impetus but considerable cognitive ambiguity. Bystanders may act because helping feels automatic, obligatory, or humanitarian. However, the same person may simultaneously experience fear, disgust, exposure, and uncertainty. Once an action begins, performance depends not only on prior knowledge but also on whether the patient can be positioned appropriately, the equipment is understandable, assistance is available, and someone coordinates the scene. The event may continue psychologically long after professional responders have departed.

This process-oriented interpretation is consistent with broader evidence that confidence, perceived competence, emotional arousal, and situational appraisal influence the initiation of CPR.^5,6,22^ However, a recent qualitative meta-synthesis examined public willingness to participate in out-of-hospital CPR,^23^ the present review demonstrates how these factors are experienced across the phases before, during, and after intervention in real OHCA events. Similarly, a scientific statement on the experiences of lay rescuers called for emergency systems to view training, on-scene responses, and residual impacts as interrelated phases.^4^

## 5.2 Recognition Should Be Trained Under Ambiguous Conditions

Public messaging often depicts cardiac arrest as a clearly recognizable sudden collapse. Synthesized evidence indicates that bystanders may encounter abnormal breathing, apparent residual movement, awkward body position, or domestic context that interferes with appraisal. Current basic life support guidelines emphasize unresponsiveness and abnormal breathing; however, training must also help the public tolerate uncertainty and understand that agonal or intermittent breathing does not indicate recovery.^2^ Scenario-based education should therefore include ambiguous presentations and explicitly reassure the public that chest compressions can be started immediately when cardiac arrest is suspected.^24–26^

## 5.3 Training Should Reproduce the Tasks of Real Resuscitation

CPR training has been identified as a key independent predictor of bystanders’ ability to promptly deliver first-aid interventions for OHCA.^27,28^The gap between training and actual events extends beyond forgetting procedural steps. Responders must also manage the patients’ body weight, floor position, rib movement, body fluids, fatigue, social hesitation, and multiple simultaneous tasks. Training should integrate realistic task transitions: recognizing abnormal breathing, moving the patient when feasible, calling emergency services on speakerphones, allocating tasks, retrieving and attaching an AED, rotating compressors, and continuing despite unpleasant sensory stimuli. Brief refresher training remains valuable, but scenario fidelity, role clarity, and teamwork practice may be as important as recall of factual knowledge.

AED design and public education should emphasize that voice prompts can transfer a part of the rescuer's cognitive burden to the device. Dispatcher guidance can likewise reduce uncertainty by describing what the bystander may observe, confirming the appropriateness of immediate action, and providing concise instructions that support on-scene leadership.

## 5.4 Post-event Care Is Part of the Emergency Response System

The emotional consequences of OHCA vary between studies. Many bystanders adjust without experiencing severe or persistent distress, whereas others experience intrusive memories, guilt, sleep disturbances, avoidance, or need for professional support. This variation indicates that post-event effects should not be ignored or universally pathologized. A stepped support pathway can include early acknowledgment, plain language information about common reactions, a mechanism for requesting an event review, communication of patient outcomes where ethically permissible, and screening or referral when distress persists. Previous research has identified uncertainty about the outcome and lack of feedback as important concerns for lay rescuers, whereas organized debriefing has been found to be valuable.^29–31^

Information sharing must comply with the patient privacy requirements and local laws. Even when a specific outcome cannot be disclosed, relevant agencies can explain what usually occurs after a patient handover, acknowledge the value of the rescue attempt, correct misconceptions regarding responsibility, and provide contact details for support. Such communication may have reduced the sense that the bystander's role ended abruptly when the ambulance departed.

## 5.5 Cultural and Contextual Considerations

The included studies spanned six countries for more than two decades. Responsibility, empathy, courage, and distress were evident across the settings. However, their expression may be shaped by training systems, dispatch practices, legal protection, family relationships, availability of AEDs, and cultural expectations of helping. Studies from mainland China and Taiwan have extended the evidence beyond Western settings; however, only two studies have represented the Asian context. Participants in the Danish study were trained bystanders in an AED volunteer network, and several studies have recruited individuals who had already acted. Such samples may have underestimated the experiences of those who did not intervene, declined participation or left the scene. Implementation should therefore be adapted locally and evaluated among populations with lower confidence, less training, or weaker links to formal emergency systems.

## 5.6 Potential Role of Digital Health Support

Although digital interventions were not directly evaluated in the included studies, the synthesized findings suggest that appropriately designed digital tools may help address uncertainty in OHCA recognition, limited access to real-time guidance, difficulties in locating AEDs, and unmet needs for post-event information and support. Telemedicine can provide health education, professional communication, and continuity of support across time and place, creating a technological basis for extending public emergency response systems across pre-event preparation, on-scene care, and post-event follow-ups.^28^ Mobile applications can deliver brief and repeated CPR/AED refresher training, present atypical OHCA scenarios, support psychological preparedness, and identify nearby AEDs. In this scene, they can integrate emergency calls, speakerphone dispatcher guidance, location services, and concise procedural prompts. After an event, secure digital platforms provide information on common psychological reactions, self-screening, routes for requesting an event review, and referral to professional support.

## 5.7 Limitations

This study has several limitations. First, the review protocol was currently registered and not publicly registered before the start of the study. To enhance transparency, the search, selection, appraisal, extraction, meta-aggregation, and ConQual grading processes have been reported. Second, the included studies varied in event setting, relationship between participants and patients, training experience, responder role, interval between the event and interview, and the depth of qualitative data. Third, the three findings from Brinkrolf et al. were not accompanied by accessible supporting illustrations because the relevant supplementary appendix cited by the original authors could not be obtained; therefore, they were rated as credible rather than unequivocal. Fourth, most participants had performed or attempted CPR, and this review may not adequately represent the experiences of those who did not. Finally, confidence was moderate for the three synthesized findings, and low for the post-event synthesized finding. Therefore, the recommendations should be evaluated during implementation rather than interpreted as definitive causal conclusions.

# 6. Conclusions

Lay bystanders are not a passive link between patient collapse and professional care. They must interpret uncertain signs, assume moral and emotional responsibilities, perform physically and socially complex tasks, and continue to experience the effects of an event long after the patient is transported. The evidence supports an integrated, rescuer-centered pathway that provides realistic preparation before an event, clear cognitive and interpersonal support during the response, and information and psychological support proportionate to the need afterward. Such a pathway may make public intervention more feasible while also recognizing the well-being of those who step forward to help.

# 7. Declarations

**Protocol and registration:** Retrospective registration was performed using the INPLASY system (INPLASY202680035).

**Data availability:** Data supporting this review are available in the included studies, tables, and accompanying appendices.

**Conflicts of interest:** The authors declare no conflicts of interest.

**Funding**: This study was supported by the Medical Research Project of the Chongqing Municipal Health Commission (Grant No.2026WSJK047); This study was supported by the Humanities and Social Sciences Fund Project of the Army Medical University (Grant No. 2022XRW21). This study was supported by the Chongqing Municipal Natural Science Foundation Key Project (Grant No. CSTB2023NSCQZDJ0016)

# References

**1**	Myat A, Song K-J, Rea T. Out-of-hospital cardiac arrest: current concepts. *The Lancet* 2018; **391**: 970–9.

**2**	Olasveengen TM, Semeraro F, Ristagno G, et al. European Resuscitation Council Guidelines 2021: Basic Life Support. *Resuscitation* 2021; **161**: 98–114.

**3**	Khan UR, Khudadad U, Baig N, et al. Out of hospital cardiac arrest: experience of a bystander CPR training program in Karachi, Pakistan. *BMC Emerg Med* 2022; **22**: 93.

**4**	Dainty KN, Colquitt B, Bhanji F, et al. Understanding the Importance of the Lay Responder Experience in Out-of-Hospital Cardiac Arrest: A Scientific Statement From the American Heart Association. *Circulation* 2022; **145**. https://doi.org/10.1161/CIR.0000000000001054.

**5**	Farquharson B, Dixon D, Williams B, et al. The psychological and behavioural factors associated with laypeople initiating CPR for out-of-hospital cardiac arrest: a systematic review. *BMC Cardiovasc Disord* 2023; **23**: 19.

**6**	Vaillancourt C, Stiell IG, Wells GA. Understanding and improving low bystander CPR rates: a systematic review of the literature. *CJEM* 2008; **10**: 51–65.

**7**	Im D, Pyo J, Lee H, Jung H, Ock M. Qualitative Research in Healthcare: Data Analysis. *J Prev Med Public Health* 2023; **56**: 100–10.

**8**	No authors listed: JBI Manual for Evidence Synthesis - JBI Manual for Evidence Synthesis - Confluence. https://jbi-global.atlassian.net/wiki/spaces/MANUAL/overview (accessed 11 Aug 2026).

**9**	Page MJ, McKenzie JE, Bossuyt PM, et al. The PRISMA 2020 statement: An updated guideline for reporting systematic reviews. *Journal of Clinical Epidemiology* 2021; **134**: 178–89.

**10**	Rethlefsen ML, Kirtley S, Waffenschmidt S, et al. PRISMA-S: an extension to the PRISMA Statement for Reporting Literature Searches in Systematic Reviews. *Syst Rev* 2021; **10**: 39.

**11**	Tong A, Flemming K, McInnes E, Oliver S, Craig J. Enhancing transparency in reporting the synthesis of qualitative research: ENTREQ. *BMC Med Res Methodol* 2012; **12**: 181.

**12**	Lockwood C, Munn Z, Porritt K. Qualitative research synthesis: methodological guidance for systematic reviewers utilizing meta-aggregation. *International Journal of Evidence-Based Healthcare* 2015; **13**: 179–87.

**13**	Munn Z, Porritt K, Lockwood C, Aromataris E, Pearson A. Establishing confidence in the output of qualitative research synthesis: the ConQual approach. *BMC Med Res Methodol* 2014; **14**: 108.

**14**	Axelsson A, Herlitz J, Fridlund B. How bystanders perceive their cardiopulmonary resuscitation intervention; a qualitative study. *Resuscitation* 2000; **47**: 71–81.

**15**	Beck S, Phillipps M, Degel A, Mochmann H-C, Breckwoldt J. Exploring cardiac arrest in ‘at-home’ settings: Concepts derived from a qualitative interview study with layperson bystanders. *Resuscitation* 2024; **194**: 110076.

**16**	Brinkrolf P, Metelmann B, Metelmann C, et al. One out of three bystanders of out-of-hospital cardiac arrests shows signs of pathological psychological processing weeks after the incident - results from structured telephone interviews. *Scand J Trauma Resusc Emerg Med* 2021; **29**: 131.

**17**	Chen H-H, Chiang W-C, Hsieh M-J, et al. Experiences and Psychological Influences in Lay Rescuers Performing Bystander Cardiopulmonary Resuscitation: A Qualitative Study. *J Acute Med* 2020; **10**: 138–48.

**18**	Malta Hansen C, Rosenkranz SM, Folke F, et al. Lay Bystanders’ Perspectives on What Facilitates Cardiopulmonary Resuscitation and Use of Automated External Defibrillators in Real Cardiac Arrests. *JAHA* 2017; **6**: e004572.

**19**	Mathiesen WT, Bjørshol CA, Braut GS, Søreide E. Reactions and coping strategies in lay rescuers who have provided CPR to out-of-hospital cardiac arrest victims: a qualitative study. *BMJ Open* 2016; **6**: e010671.

**20**	Mausz J, Snobelen P, Tavares W. “Please. Don’t. Die.”: A Grounded Theory Study of Bystander Cardiopulmonary Resuscitation. *Circ: Cardiovascular Quality and Outcomes* 2018; **11**: e004035.

**21** Yang L, Li B, Zeng Y, Cai SQ, Zhou M, Zhang H. Qualitative Study on the Resuscitation Experience of First Responders to Cardiopulmonary Resuscitation. Chinese Journal of Emergency Resuscitation and Disaster Medicine. 2022;17(4):430-434. Accessed January 12, 2026.

**22**	Sasson C, Haukoos JS, Bond C, et al. Barriers and Facilitators to Learning and Performing Cardiopulmonary Resuscitation in Neighborhoods With Low Bystander Cardiopulmonary Resuscitation Prevalence and High Rates of Cardiac Arrest in Columbus, OH. *Circ: Cardiovascular Quality and Outcomes* 2013; **6**: 550–8.

**23**	Cheng Y, Zhang C, Chen L, Liu H, He W, Shi Z. Public participation willingness in out-of-hospital cardiopulmonary resuscitation: A systematic review and meta-analysis. *Int J Nurs Sci* 2025; **12**: 192–9.

**24**	Bobrow BJ, Spaite DW, Berg RA, et al. Chest Compression–Only CPR by Lay Rescuers and Survival From Out-of-Hospital Cardiac Arrest.

**25**	Shende TC, Battaglia MR, Nuno T, Beskind D. Efficacy of a five-minute compression-only cardiopulmonary resuscitation class compared to thirty-minute instruction among college students. *Resusc Plus* 2020; **3**: 100012.

**26**	Iwami T, Kitamura T, Kawamura T, et al. Chest Compression–Only Cardiopulmonary Resuscitation for Out-of-Hospital Cardiac Arrest With Public-Access Defibrillation: A Nationwide Cohort Study. *Circulation* 2012; **126**: 2844–51.

**27**	Hayıroğlu Mİ. Telemedicine: Current Concepts and Future Perceptions. *Anatol J Cardiol* 2019. https://doi.org/10.14744/AnatolJCardiol.2019.12525.

**28**	Hayıroğlu Mİ, Çınar T, Çinier G, et al. The effect of 1-year mean step count on the change in the atherosclerotic cardiovascular disease risk calculation in patients with high cardiovascular risk: a sub-study of the LIGHT randomized clinical trial. *Kardiol Pol* 2021; **79**: 1140–2.

**29**	Mathiesen WT, Bjørshol CA, Høyland S, Braut GS, Søreide E. Exploring How Lay Rescuers Overcome Barriers to Provide Cardiopulmonary Resuscitation: A Qualitative Study. *Prehospital and Disaster Medicine* 2017; **32**: 27–32.

**30**	Zijlstra JA, Beesems SG, Haan RJD, Koster RW. Psychological impact on dispatched local lay rescuers performing bystander cardiopulmonary resuscitation. *Resuscitation* 2015; **92**: 115–21.

**31**	Møller TP, Hansen CM, Fjordholt M, Pedersen BD, Østergaard D, Lippert FK. Debriefing bystanders of out-of-hospital cardiac arrest is valuable. *Resuscitation* 2014; **85**: 1504–11.

**Table, and Appendix Captions**

• Table 1. Characteristics of the included studies (N = 8).

• Table 2. Results of the JBI methodological quality appraisal.

• Table 3. Meta-aggregation of synthesized findings, categories, original authors' findings, and credibility levels.

• Table 4. Summary of the findings developed using the ConQual approach.

Appendix I. Complete Search Strategies.

• Appendix II. Extraction table of original authors' findings, illustrations, source page numbers, and credibility levels.

**Table 1. Characteristics of included studies (N = 8)**

| **Author (year), country** | **Study design** | **Participants**  **(sample=n)** | **Context / setting** | **phenomenon of interest** | **data collection** | **Data analysis** |
| --- | --- | --- | --- | --- | --- | --- |
| Axelsson et al.^14^ (2000) Sweden | Qualitative study | Bystanders who performed CPR during an OHCA  (n = 19) | Sweden; out-of-hospital cardiac arrest events involving lay bystanders | To describe how bystanders perceived their CPR intervention. | Individual, tape-recorded interviews guided by a question template/interview guide. | Phenomenographic analysis; findings organized into five main categories and 14 subcategories. |
| Beck et al.^15^ (2024) Germany | Qualitative interview study | Lay witnesses of at-home cardiac arrest  (n = 21) | Berlin, Germany; cardiac arrests in patients’ homes | To explore factors facilitating and impeding B-CPR in at-home cardiac arrest and derive overarching concepts. | Face-to-face semi-structured interviews at the participant’s home or a quiet hospital room | Qualitative content analysis according to Mayring; category system, inductive derivation of six concepts. |
| Brinkrolf et al.^16^ (2021) Germany | Post-hoc analysis of a semi-standardized telephone-interview dataset | Adult-OHCA witnesses  ( n = 89) | Münster, Germany; witnessed adult OHCA from Dec 2014 to Apr 2016 | To examine bystanders’ psychological processing after OHCA and its associations with performing CPR and with relationship to the patient. | A semi-standardized telephone questionnaire included an open question about the paramount emotion; responses were recorded verbatim. | Two independent raters categorized responses. |
| Chen et al.^17^ (2020) Taiwan,China | Grounded theory | Lay rescuers from 5 events  ( n = 9) | Taiwan; public locations and the national Public AED Registry/PAD program | To characterize experiences surrounding bystander-initiated resuscitation and psychological influences after the event in an Asian community. | Individual, in-depth, face-to-face semi-structured interviews using an 18-question outline; audio-recorded. | Content analysis of verbatim transcripts; coding into categories and subcategories. Two researchers analyzed independently and reached consensus. |
| Malta Hansen et al.^18^ (2017) Denmark | Qualitative study | 128 lay bystanders were interviewed. Twenty-six recorded interviews were purposively selected for in-depth qualitative analysis  (n = 26) | Denmark; authentic OHCAs involving a donated-AED network | To identify factors facilitating lay bystander CPR and AED use in authentic OHCA. | semi-structured telephone interviews; recruitment/selection continued to data saturation. | Five-stage iterative, inductive cross-sectional indexing. |
| Mathiesen et al. ^19^ (2016) Norway | Exploratory qualitative study | Lay rescuers from 18 OHCA events  (n = 20) | Stavanger region, Norway; event-to-interview interval 6 days–13 years | To explore reactions and coping strategies among lay rescuers after providing CPR to OHCA victims. | 18 face-to-face and 2 telephone in-depth semi-structured interviews; audio-recorded. | Inductive qualitative content analysis (*Graneheim and Lundman*): meaning units, codes, subcategories, three categories and one overarching theme. |
| Mausz et al.^20^ (2018) Canada | Constructivist grounded theory (*Charmaz*) | Lay rescuers who had recently intervened in an OHCA  ( n = 15) | Ontario, Canada; participants recruited through Peel Regional Paramedic Services following recent OHCA interventions. | To explore bystander CPR and develop a mid-range explanatory theory of the lay-rescuer process. | One individual interview and five focus groups (2–7 participants), semi-structured, 45–90 minutes. | Constant comparative analysis with theoretical sampling until theoretical saturation; development of a three-stage explanatory model. |
| Yang Lu et al.^21^ (2022) China | Phenomenological qualitative study | Bystanders with experience of performing CPR  ( n = 10) | Hainan, China; one-to-one interviews conducted in an independent, quiet setting. | To explore first responders’ experiences of performing CPR. | One-to-one semi-structured in-depth interviews (about 40 minutes), audio-recorded with field notes. | Colaizzi’s seven-step phenomenological analysis using NVivo 11. |

- Notes:AED, automated external defibrillator; B-CPR, bystander cardiopulmonary resuscitation; CPR, cardiopulmonary resuscitation; EMS, emergency medical services; OHCA, out-of-hospital cardiac arrest; PAD, public access defibrillation.

**Table 2. Results of Study Quality Assessment**

| **Study** | **（1）** | **（2）** | | **（3）** | | **（4）** | **（5）** | **（6）** | **（7）** | **（8）** | **（9）** | **（10）** | | **Score** |
| --- | --- | --- | --- | --- | --- | --- | --- | --- | --- | --- | --- | --- | --- | --- |
| Axelsson et al.^14^ (2000) | Y | | Y | | Y | Y | Y | N | U | Y | Y | Y | 8/10 | |
| Beck et al.^15^ (2024) | Y | | Y | | Y | Y | Y | N | N | Y | Y | Y | 8/10 | |
| Brinkrolf et al.^16^ (2021) | N | | Y | | Y | Y | Y | N | N | Y | Y | Y | 7/10 | |
| Chen et al.^17^ (2020) | Y | | Y | | Y | Y | Y | Y | U | Y | Y | Y | 9/10 | |
| Malta Hansen et al.^18^ (2017) | U | | Y | | Y | Y | Y | Y | Y | Y | Y | Y | 9/10 | |
| Mathiesen et al. ^19^ (2016) | U | | Y | | Y | Y | Y | Y | U | Y | Y | Y | 8/10 | |
| Mausz et al.^20^ (2018) | Y | | Y | | Y | Y | Y | Y | Y | Y | Y | Y | 10/10 | |
| Yang Lu et al.^21^ (2022) | U | | Y | | Y | Y | Y | N | U | Y | Y | Y | 7/10 | |

Note: Y=Yes; N=No; U=Unclear

(1): Is there congruity between the stated philosophical perspective and the research methodology?
(2): Is there congruity between the research methodology and the research question or objectives?
(3): Is there congruity between the research methodology and the methods used to collect data?
(4): Is there congruity between the research methodology and the representation and analysis of data?
(5): Is there congruity between the research methodology and the interpretation of results?
(6): Is there a statement locating the researcher culturally or theoretically?
(7): Is the influence of the researcher on the research, and vice versa, addressed?
(8): Are participants, and their voices, adequately represented?
(9): Is the research ethical according to current criteria or, for recent studies, is there evidence of ethical approval by an appropriate body?
(10): Do the conclusions drawn in the research report flow from the analysis, or interpretation, of the data?

**Table 3. Meta-aggregation of synthesized findings, categories, and original-author findings with level of credibility**

| **Synthesized finding** | **Category** | **Study findings** |
| --- | --- | --- |
| SF1  Bystander action is motivated by prosocial responsibility and automatic helping, but may be inhibited by perceived risks, repugnance, and psychological burden. | Category 1.1  Prosocial motivation, responsibility, courage, and automatic action | A1 (U) — To have a sense of humanity (to wish to save lives; to wish to help; to act intuitively).  A3 (U) — To feel an obligation (to take responsibility; to do one's duty).  A4 (U) — To have courage (to dare; to have an intrinsic power).  B6 (U) — Automaticity of actions.  C1 (U) — Motivation: The rescuers showed helping traits, individual cognition, and situation awareness, which were factors motivating the resuscitation efforts.  Y1 (U) — First responders had a strong sense of responsibility and empathy. |
|  | Category 1.2  Exposure, repugnance, psychological burden, and perceived risk | A5 (U) — To feel exposed (to feel deserted; powerless; ambivalent; uncertain; and repugnance).  Y4 (U) — First responders experienced a heavy psychological burden during CPR and found it difficult to regulate. |
| SF2  Unexpected OHCA presentations and misinterpreted signs delay recognition of cardiac arrest and the perceived need for immediate CPR. | Category 2.1  Unexpectedness and difficulty in situational judgement | B1 (U) — Unexpectedness of the event.  B3 (U) — Situational judgement. |
|  | Category 2.2  Misinterpretation of signs and failure to recognize the need for CPR | B4 (U) — Awareness of the necessity to perform B-CPR.  MA2 (U) — Taking action to save the victim is complicated by misconceptions about cardiac arrest and by practical CPR/AED challenges. |
| SF3  Hands-on training, workable conditions, usable equipment, teamwork, and leadership facilitate resuscitation; limited preparedness and the training–reality gap impede performance.  SF3 (continued) | Category 3.1  Preparedness, prior training, and the training–reality gap | A2 (U) — To have competence (to have knowledge; to feel prepared).  C2 (U) — Training-reality discrepancies: Rescuers reported discrepancies between actual resuscitation, prior training experiences, and their expectations.  H1 (U) — Knowledge and skills from CPR courses: Previous hands-on CPR training and knowledge that intervention is vital and cannot cause substantial harm facilitated action.  Y2 (U) — First responders' first-aid skills and competence needed improvement.  Y3 (U) — First responders lacked professional first-aid knowledge and literacy. |
|  | Category 3.2  Physical, environmental, equipment, and technical conditions | B5 (U) — Initial position of the patient.  H2 (U) — Ventilation masks: A mask helped bystanders overcome oral fluids and repugnance during ventilation.  H3 (U) — The AED apparatus: Familiarity with the AED, prior knowledge that it would guide CPR, and its voice prompts increased reassurance and confidence. |
|  | Category 3.3  Teamwork and on-scene leadership | H4 (U) — Bystander teamwork: Division and delegation of tasks helped the resuscitation attempt run effectively.  H5 (U) — Leadership and the moral imperative to act: Showing leadership and feeling a moral obligation drove participation. |
| SF4  Responding to OHCA may cause acute or persistent distress; recovery is shaped by psychological processing, patient outcomes, information, support, and meaning-making. | Category 4.1  Acute stress, panic, and shock | B2 (U) — Acute stress response.  MA1 (U) — Being called to act is disturbing, causing panic, shock, and disbelief that must ultimately be overcome. |
|  | Category 4.2  Persistent distress, self-blame, and difficulty making sense of the event | BR1 (C) — Signs of pathological psychological processing.  M1 (U) — Concern.  MA3 (U) — Making sense of the experience is challenging: lay rescuers contend with self-doubt, unanswered questions, and uncomfortable emotional reactions. |
|  | Category 4.3  Physiological psychological processing and limited or no distress | BR2 (C) — Physiological psychological processing.  BR3 (C) — No signs of psychological distress due to the OHCA. |
|  | Category 4.4  Outcome-dependent adjustment, recovery, and personal growth | C3 (U) — Psychological influence: Reflection on the event involved mental strengthening, greater responsibility and confidence; short-term negative reactions also occurred, and rescuers remained willing to help in future.  Y5 (U) — CPR outcomes had a profound impact on first responders' quality of life. |
|  | Category 4.5  Coping, support, and access to patient-outcome information | M2 (U) — Uncertainty.  M3 (U) — Coping strategies. |

Note: 1.U = unequivocal; C = credible; NS = not supported. Only U and C findings are included in the meta-aggregation.

2.AED, automated external defibrillator; B-CPR, bystander cardiopulmonary resuscitation; CPR, cardiopulmonary resuscitation; OHCA, out-of-hospital cardiac arrest;

**Table 4. Summary of Findings (ConQual)**

| **Systematic review title** | Lay bystanders’ experiences and perceptions of responding to out-of-hospital cardiac arrest: a qualitative systematic review | | | | | |
| --- | --- | --- | --- | --- | --- | --- |
| **Population** | Lay bystanders and first responders who witnessed an OHCA, including those who initiated, attempted, or considered CPR and/or AED use. | | | | | |
| **Interest of phenomenon** | Experiences and perceptions surrounding recognition of OHCA, decisions to act, real-world resuscitation, and post-event psychological impact. | | | | | |
| **Context** | OHCA events in community, public, workplace, and home settings across Sweden, Germany, Taiwan, Denmark, Norway, Canada, and China. | | | | | |
| **Synthesized finding** | | **Type of research** | **Dependability** | **Credibility** | **ConQual level** | **Comments** |
| SF1 Bystander action is motivated by prosocial responsibility and automatic helping, but may be inhibited by perceived risks, repugnance, and psychological burden. | | Qualitative | Downgrade 1 level | No change | Moderate | **Dependability:** Downgraded 1 level due to methodological limitations across the included primary studies.  **Credibility:** no change; 8 U, 0 C. |
| SF2 Unexpected OHCA presentations and misinterpreted signs delay recognition of cardiac arrest and the perceived need for immediate CPR. | | Qualitative | Downgrade 1 level | No change | Moderate | **Dependability:** Downgraded 1 level due to methodological limitations across the included primary studies.  **Credibility:** no change; 4 U, 0 C. |
| SF3 Hands-on training, workable conditions, usable equipment, teamwork, and leadership facilitate resuscitation; limited preparedness and the training–reality gap impede performance. | | Qualitative | Downgrade 1 level | No change | Moderate | **Dependability:** Downgraded 1 level due to methodological limitations across the included primary studies.  **Credibility:** no change; 10 U, 0 C. |
| SF4 Responding to OHCA may cause acute or persistent distress; recovery is shaped by psychological processing, patient outcomes, information, support, and meaning-making. | | Qualitative | Downgrade 1 level | Downgrade 1 level | Low | **Dependability:** Downgraded 1 level due to methodological limitations across the included primary studies.  **Credibility:** Downgraded 1 level due to a mix of unequivocal (U) and credible (C) findings; 8 U and 3 C. |

Note: 1.U = unequivocal; C = credible; NS = not supported. Only U and C findings are included in the meta-aggregation.

2.AED, automated external defibrillator; CPR, cardiopulmonary resuscitation; OHCA, out-of-hospital cardiac arrest.
